# EXAMINING THE IMPACT OF POSTSTROKE COGNITIVE IMPAIRMENT ON FUNCTIONAL MOBILITY

**DOI:** 10.1093/geroni/igae098.3315

**Published:** 2024-12-31

**Authors:** Anjali Tiwari, Neha Lodha

**Affiliations:** Colorado State University, Fort Collins, Colorado, United States; Colorado State University, Fort Collins, Colorado, United States

## Abstract

Approximately, 70% of individuals experience some degree of cognitive impairment following stroke. The study aims to determine the impact of post-stroke cognitive impairment on functional mobility.

**Methods:**

Twenty-nine stroke survivors (cognitively impaired; N = 15 and cognitively normal; N = 14) participated in this study. Participants performed (1) Overground walking while simultaneously performing a secondary cognitive task. We measured gait speed (m/s) and stride time variability (%). 2) Simulator driving task: Participants performed a car-following task and responded to the brake lights of the lead car. We measured the mean (pounds), and variability in brake force (%). To examine whether post-stroke cognitive impairment influences performance on walking and driving tasks, we conducted a one-way ANCOVA test while adjusting for the stroke motor severity assessed by the Modified Rankin Scale. Results – The cognitively impaired stroke group showed slower gait speed (F(1, 26) =9.594, p=0.005), increased stride length variability (F(1, 26) =5.505, p=0.033), and brake force variability (F(1, 26) =3.316, p= 0.035) compared with the cognitively normal stroke group. The two groups showed no significant difference in mean brake force (F (1, 26) = 3.345 p= 0.087). Conclusion- Post-stroke cognitive impairment significantly impacts functional mobility and contributes to impaired walking and braking function, even after controlling for the severity of motor impairment post-stroke. Significance- The findings underscore the critical role of cognitive function in post-stroke recovery of functional mobility, above and beyond motor function. Intervention targeting cognitive function alongside motor function would be important for optimal functional recovery following stroke.

